# Chronic Disease Patterns and Their Relationship With Health-Related Quality of Life in South Korean Older Adults With the 2021 Korean National Health and Nutrition Examination Survey: Latent Class Analysis

**DOI:** 10.2196/49433

**Published:** 2024-04-10

**Authors:** Mi-Sun Lee, Hooyeon Lee

**Affiliations:** 1 Department of Preventive Medicine College of Medicine The Catholic University of Korea Seoul Republic of Korea

**Keywords:** chronic disease, latent class analysis, multimorbidity, older adults, quality of life

## Abstract

**Background:**

Improved life expectancy has increased the prevalence of older adults living with multimorbidities, which likely deteriorates their health-related quality of life (HRQoL). Understanding which chronic conditions frequently co-occur can facilitate person-centered care tailored to the needs of individuals with specific multimorbidity profiles.

**Objective:**

The study objectives were to (1) examine the prevalence of multimorbidity among Korean older adults (ie, those aged 65 years and older), (2) investigate chronic disease patterns using latent class analysis, and (3) assess which chronic disease patterns are more strongly associated with HRQoL.

**Methods:**

A sample of 1806 individuals aged 65 years and older from the 2021 Korean National Health and Nutrition Examination Survey was analyzed. Latent class analysis was conducted to identify the clustering pattern of chronic diseases. HRQoL was assessed by an 8-item health-related quality of life scale (HINT-8). Multiple linear regression was used to analyze the association with the total score of the HINT-8. Logistic regression analysis was performed to evaluate the odds ratio of having problems according to the HINT-8 items.

**Results:**

The prevalence of multimorbidity in the sample was 54.8%. Three chronic disease patterns were identified: relatively healthy, cardiometabolic condition, arthritis, allergy, or asthma. The total scores of the HINT-8 were the highest in participants characterized as arthritis, allergy, or asthma group, indicating the lowest quality of life.

**Conclusions:**

Current health care models are disease-oriented, meaning that the management of chronic conditions applies to a single condition and may not be relevant to those with multimorbidities. Identifying chronic disease patterns and their impact on overall health and well-being is critical for guiding integrated care.

## Introduction

The prevalence of chronic conditions among older populations is growing due to the progressive increase in life expectancy [1]. Multimorbidity, defined as the co-occurrence of 2 or more chronic conditions, continues to increase worldwide, presenting one of today’s major challenges to health at the individual and population levels [1,2]. Certain chronic diseases tend to co-occur more often than expected by chance because they share pathophysiological pathways [2,3].

One systematic review reported multimorbidity patterns, especially cardiometabolic conditions, mental health issues, and musculoskeletal disorders [4]. Another study showed 3 multimorbidity groups: cardiovascular and metabolic diseases, mental health problems, and allergic diseases [5,6]. Hypertension, dyslipidemia, stroke, and diabetes mellitus are highly likely to co-occur [2], with hypertension having stronger connections with hyperlipidemia and diabetes than other pairs of morbidities in Korean older adults [2]. Health-related quality of life (HRQoL) is an important health outcome indicator in the aging process. Previous observational studies have shown the negative effects of multimorbidity on HRQoL [7,8].

While multimorbidity studies have applied a variety of methodologies, such as cluster analysis, factor analysis, and latent class analysis (LCA), none of these approaches enable direct comparisons. LCA is preferred to conventional clustering because it uses probability-based classification methods to select an optimal number of classes based on various diagnostic tests [3,9], which allows researchers to group individuals into a number of latent classes and then analyze the differences between those classes [10].

South Korea, which became an aged society in 2017, is the world’s fastest aging country, with more than 14% of its population aged 65 years and older [6]. Further, among Korean adults aged 50 years and older, 39% have 2 or more chronic diseases [3]. However, few studies have applied LCA to extract the multimorbidity patterns among Korean older adults. Understanding which chronic conditions frequently co-occur can facilitate person-centered care tailored to the needs of individuals with specific profiles of multimorbidity [7,8,11,12]. One of the first steps is to identify the prevalence and distinct patterns of multimorbidity to inform clinical guidelines and facilitate integrated care [9,10]. Chronic conditions tend to cluster together into multimorbidity patterns. However, further replicability of results between studies that use different methodologies is an important step toward moving from exploratory to confirmatory approaches.

Therefore, in this study, we aimed to (1) examine the prevalence of multimorbidity among Korean older adults, (2) investigate chronic disease patterns using LCA, and (3) assess which chronic disease patterns are more strongly associated with HRQoL using data taken from the 2021 Korean National Health and Nutrition Examination Survey (KNHANES).

## Methods

### Data and Study Population

The KNHANES is conducted annually to assess the health and health-related behaviors of the Korean population based on Article 16 of the National Health Promotion Act. It collects comprehensive data on the entire Korean population’s sociodemographic characteristics, health behaviors, health status, biochemical profiles, disease history, and nutrient intake [4,5]. A stratified, multistage probability sampling method was used. Participants included Korean family members aged 1 year and older in the selected households, corresponding to 9682 individuals. The health interview and health examination surveys were conducted in mobile examination centers, while the nutrition survey was performed by visiting households [6]. Of the 9682 eligible participants, 7090 (response rate=73.2%) ultimately participated in the survey. Of these survey respondents, we finally selected 1806 individuals aged 65 years and older.

### Measurements

#### Chronic Diseases

Multimorbidity is indicated by the presence of 2 or more of the diseases in a single individual. The diagnosis of chronic conditions was based on whether the respondent had ever been diagnosed with a chronic disease by a doctor and received treatment for that disease [3,7,8]. When the prevalence of multimorbidity is low, the sample size for certain combinations of multimorbid conditions may be insufficient to provide reliable results [9]. Therefore, our measurement of multimorbidity was limited to 9 chronic conditions with a prevalence of at least 3% among the older people measured in the KNHANES [10,11]: hypertension, dyslipidemia, myocardial infarction, stroke, diabetes mellitus, arthritis, osteoarthritis, asthma, or allergic rhinitis.

#### Health-Related Quality of Life

The HRQoL was assessed by the 8-item health-related quality of life scale (HINT-8), an instrument developed in 2014 as a quality-of-life scale reflective of Korean culture [5]. The items on the HINT-8 are difficulty climbing stairs, pain, lack of vitality, difficulty working, depression, difficulty in memory, sleep problems, and unhappiness [12]. Each question is rated at 4 levels (none, mild, moderate, and severe problems). The total score ranges from 4 to 32 points. Higher scores on the HINT-8 indicate a poorer quality of life. These 4 levels were divided into one group having “no problems” and the other having “problems” (mild, moderate, and severe problems) [13-15]. In this study, the Cronbach α coefficient of the HINT-8 indicated satisfactory internal consistency (α=0.817).

#### Covariates

The covariate variables were age, sex, household income, education level, employment status, marital status, current smoking status, and current drinking status. Household income included wages, pensions, bank interest, social security benefits, and unemployment benefits [16]. Marital status was classified as married, cohabiting, divorced, bereaved, or separated. Current alcohol consumption was defined as adults who consumed alcohol at least once in the previous month [4,17]. Current smoking was defined as an adult with a lifetime smoking history of 5 packs or more who also currently smokes [17]. Household income was defined as the monthly average gross income divided by an equivalence factor to adjust for differences in household size and composition [18] and was categorized into quintiles. This study reclassified the quintiles into 3 groups (upper 2 quintiles, middle 2 quintiles, and lower 2 quintiles). Education level was categorized as middle school graduation or below, high school graduation, and college graduation or above. Employment status was categorized as employed or unemployed.

### Statistical Analysis

#### Latent Class Analysis

LCA is a statistical procedure used to identify qualitatively different subgroups within populations who often share certain outward characteristics [19]. Subgroups are referred to as latent groups (or classes). To detect latent groups, LCA uses participants’ responses to categorical variables. Individuals with similar chronic disease patterns are then classified into distinct subgroups, and a label is derived for each pattern based on the salient characteristics [20]. This special case of person-centered mixture modeling can thus identify the latent subpopulations within a sample based on patterns of responses to observed variables [19].

LCA was conducted using the R-based Jamovi (version 2.3.24; The Jamovi Project). Models with 1-5 classes were estimated. The likelihood ratio statistic (G^2^), Akaike information criterion, Bayesian information criterion, and entropy value (0.0-1.0, ≥0.70 is acceptable) were used to identify the optimal number of classes [21]. The final selection model considered class distinction, plot interpretability, and estimated sample size. The multiple fit statistics indicated that a 3-class model was the best fit for this study’s data (G^2^=–522, Akaike information criterion 14,352, Bayesian information criterion 14,604, entropy 0.876; *P*=.01; Table S1 and Figure S1 in Multimedia Appendix). These 3 classes were called the relatively healthy group (class 1), the cardiometabolic condition group (class 2), and the arthritis, allergy, or asthma group (class 3).

#### Association Analysis

After determining the 3 latent classes, descriptive statistics, analysis of variance, and chi-square tests were used to analyze the prevalence distribution of the participants’ sociodemographic characteristics and HRQoL. Multiple linear regression analyses were conducted to assess the association between the contribution of the latent classes and the total score of the HINT-8 after adjusting for age, sex, household income, education level, marital status, employment status, current smoking status, and current drinking status.

Multiple logistic regression analyses were conducted separately for each HINT-8 subfactor (difficulty in climbing stairs, pain, lack of vitality, difficulty working, depression, memory difficulties, sleep problems, and unhappiness). The odds ratios (ORs) and 95% CIs were calculated after adjusting for the covariates. A multistage cluster sampling design was adopted using the SPSS Complex Sample, SPSS/WIN (version 25.0; IBM Corp) program.

### Ethical Considerations

The 2021 KNHANES was approved by the institutional review board (IRB) of the Korea Centers for Disease Control and Prevention (2018-01-03-5C-A). This study used publicly available secondary, deidentified data. The IRB review exemption was approved by the IRB of the Catholic University of Korea.

## Results

### Chronic Disease Patterns

**Figure 1:**
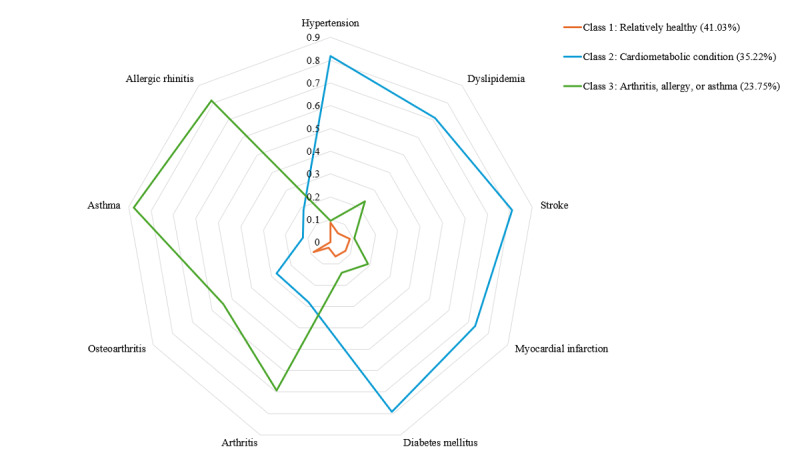
A 3-class model: estimated probabilities of belonging to the latent class.

Table 1 presents the estimated probabilities of belonging to the 3 classes. Class 1 patients had the lowest probability of having any of the 9 chronic diseases. Class 2 patients had a high probability of having hypertension, dyslipidemia, diabetes mellitus, myocardial infarction, and stroke. Class 3 patients had a high probability of having arthritis, osteoarthritis, allergic rhinitis, or asthma. Figure 1 shows the distribution of the three latent classes according to the 9 chronic diseases.

Table 2 presents the sociodemographic characteristics, chronic disease patterns, and HRQoL according to the latent classes. Of this study’s sample of 1806 Korean older adults, 42.7% (771/1806) were male participants. The mean age was 73.36 (SD 5.18) years. The prevalence of multimorbidity in the sample was 54.8%. The mean HRQoL measured by the HINT-8 was 14.07 (SD 3.89).

Among the 3 latent classes, class 1 (741/1806, 41.03%) had the lowest prevalence of current smoking and drinking. The mean number of chronic diseases was 0.57 (SD 0.59). The class 2 (636/1806, 35.22%) group with cardiometabolic conditions had a mean 2.65 (SD 1.06) chronic diseases per individual. Among them, 71.9% (447/622) experienced pain, 67.2% (418/622) lacked vitality, 44.8% (278/620) had depression, and 74% (459/620) felt unhappy. Of the class 3 group (429/1806, 23.75%) that included individuals with arthritis, allergies, or asthma, 79.8% (340/426) of the participants reported difficulty in climbing stairs, 81.2% (346/426) reported pain, 55.2% (342/426) reported depression, and 64.6% (275/426) reported sleep problems. The mean number of chronic diseases in class 3 was 2.50 (SD 1.13). Compared with class 1 participants, those participants classified into classes 2 and 3 were older, more likely to be female participants, and had lower levels of education.

**Table 1:** A 3-class model: estimated probabilities of belonging to each latent class.

Characteristics		Class 1: relatively healthy		Class 2: cardiometabolic condition		Class 3: arthritis, allergy, or asthma			
Probability of belonging to the class (%)		0.44		0.32		0.24			
**Item response probabilities (%)**									
	Hypertension		0.09		0.82^a^		0.10		
	Dyslipidemia	0.05		0.71^a^		0.23			
	Diabetes mellitus	0.07		0.79^a^		0.14			
	Myocardial infarction	0.08		0.73^a^		0.19			
	Stroke	0.09		0.81^a^		0.11			
	Arthritis	0.03		0.28		0.69^a^			
	Osteoarthritis	0.19		0.27		0.54^a^			
	Allergic rhinitis	0.00		0.19		0.81^a^			
	Asthma	0.00		0.12		0.88^a^			

^a^These indices had the highest probabilities.

**Table 2:** Sociodemographic characteristics, chronic disease patterns, and mental health by latent class.

Characteristics						Total		Class 1		Class 2		Class 3		*P* value	
Total, n (%)						1806 (100)		741 (41.03)		636 (35.22)		429 (23.75)		N/A^a^	
Age (years), mean (SD)						73.36 (5.18)		73.36 (5.42)		73.56 (4.96)		73.08 (5.08)		.29^b^	
**Sex, n (%)**															<.001
		Male			771 (42.7)		401 (54.1)		279 (43.9)		91 (21.2)				
		Female			1035 (57.3)		340 (45.9)		357 (56.1)		338 (78.8)				
**Household income, n (%)**															.35
		High			135 (7.6)		66 (9.1)		42 (6.7)		27 (6.3)				
		Middle			999 (55.9)		406 (55.7)		356 (56.4)		237 (55.5)				
		Low			653 (36.5)		257 (35.3)		233 (36.9)		163 (38.2)				
**Education level, n (%)**															<.001
		≥College			199 (12.3)		98 (16.1)		67 (11.5)		34 (8)				
		High school			325 (20.1)		128 (21.1)		128 (22.0)		69 (16.3)				
		≤Middle school			1089 (67.5)		381 (62.8)		387 (66.5)		321 (75.7)				
**Employment status, n (%)**															.001
	Employed				655 (40.5)		283 (46.5)		217 (37.2)		155 (36.5)				
	Unemployed				961 (59.5)		325 (53.5)		366 (62.8)		270 (63.5)				
**Marital status, n (%)**															.002
		Married or cohabiting			1174 (65)		513 (69.3)		407 (64)		254 (59.2)				
		Divorced, bereaved, or separated			631 (35)		227 (30.7)		229 (36)		175 (40.8)				
**Current smoking status, n (%)**															.21
			Smoker			166 (25.1)		89 (27.7)		52 (21.3)		25 (26)			
			Nonsmoker			495 (74.9)		232 (62.3)		192 (78.7)		71 (74)			
**Current drinking status, n (%)**															.01
				Drinker		768 (60.3)		348 (65)		258 (56.8)		162 (57)			
				Nondrinker		505 (39.7)		187 (35)		196 (43.2)		122 (43)			
**Multimorbidity (≥2 chronic diseases), n (%)**															<.001
			Yes			892 (54.8)		0 (0)		517 (88.5)		349 (81.4)			
			No			736 (45.2)		429 (100)		67 (11.5)		80 (18.6)			
Number of diagnosed chronic diseases, mean (SD)						1.82 (1.35)		0.57 (0.59)		2.65 (1.06)		2.50 (1.13)		<.001^b^	
**Chronic disease, n (%)**															<.001
	Hypertension				983 (54.6)		258 (35.1)		527 (82.9)		198 (46.2)				
	Dyslipidemia				610 (33.9)		35 (4.8)		417 (65.6)		158 (36.8)				
	Diabetes mellitus				408 (22.7)		41 (5.6)		307 (48.3)		60 (14)				
	Myocardial infarction				118 (7.2)		12 (2)		71 (12.2)		35 (8.2)				
	Stroke				66 (3.7)		5 (0.7)		55 (8.6)		6 (1.4)				
	Arthritis				444 (27.3)		30 (4.9)		140 (24)		274 (63.9)				
	Osteoarthritis				295 (16.4)		16 (2.2)		69 (10.8)		210 (49)				
	Allergic rhinitis				90 (5)		4 (0.5)		35 (5.5)		51 (11.9)				
	Asthma				66 (3.7)		0 (0)		14 (2.2)		52 (12.1)				
Health-related quality of life (HINT-8^c^), mean (SD)						14.07 (3.89)		14.48 (4.24)		15.62 (4.42)		16.78 (4.33)		.005^b^	
**HINT-8, n (%)**															<.001
		Difficulty in climbing stairs			1195 (68.2)		422 (59.9)		433 (69.5)		340 (79.8)				
		Pain			1220 (69.6)		427 (60.7)		447 (71.9)		346 (81.2)				
		Lack of vitality			1176 (67.3)		430 (61.3)		418 (67.2)		328 (77.2)				
		Difficulty in working			1143 (65.2)		417 (59.1)		421 (67.7)		305 (71.8)				
		Depression			786 (44.9)		273 (38.7)		278 (44.8)		342 (55.2)				
		Difficulty in memory			1197 (68.4)		460 (65.2)		410 (66.1)		327 (76.8)				
		Sleep problems			957 (54.6)		338 (47.9)		344 (55.3)		275 (64.6)				
		Unhappiness			1261 (72.1)		465 (66.2)		459 (74)		337 (79.1)				

^a^N/A: not applicable.

^b^ANOVA was performed for the continuous variables.

^c^HINT-8: 8-item health-related quality of life instrument.

### Associations Between Multimorbidity Classes and HRQoL

Table 3 presents the associations among the 3 latent classes and the total scores of the HINT-8. Class 2 patients had higher HRQoL scores by 1.14 points than class 1. Class 3 patients had higher HRQoL scores by 2.30 points than class 1 patients, which was significant.

Table 4 presents the adjusted ORs and shows the associations between chronic disease patterns and HRQoL based on the HINT-8 items. The class 2 group had a higher OR of difficulty in climbing stairs (OR 1.49, 95% CI 1.08-2.06), pain (OR 1.58, 95% CI 1.13-2.23), and unhappiness (OR 1.28, 95% CI 1.03-1.69) than the class 1 group. The class 3 group had a higher OR of difficulty in climbing stairs (OR 1.92, 95% CI 1.35-2.71), pain (OR 1.88, 95% CI 1.32-2.67), lack of vitality (OR 1.35, 95% CI 1.02-1.79), depression (OR 2.51, 95% CI 1.09-4.98), sleep problems (OR 1.46, 95% CI 1.01-2.10), and unhappiness (OR 1.54, 95% CI 1.13-2.26) than the class 1 group. Generally, the class 3 group was more likely to have problems in all 8 items of the HINT-8, even in the absence of a statistically significant result for the lack of vitality and memory difficulties.

**Table 3:** Association between chronic disease patterns and the total scores of the 8-item health-related quality of life instrument according to multiple linear regression analysis.

Latent class model	Estimate	SE	*t* test (*df*)	*P* value	Fit
(Intercept)	14.48	0.16	88.17 (1736)	<.001	*R*^2^=0.174^a^
Class 1 (relatively healthy)	Reference	N/A^b^	N/A	N/A	N/A
Class 2 (cardiometabolic conditions)	1.14	0.24	4.75 (1736)	<.001	N/A
Class 3 (arthritis, allergy, or asthma)	2.30	0.27	8.61 (1736)	<.001	N/A

^a^*P*<.001.

^b^N/A: not applicable.

**Table 4:** Association between chronic disease patterns and the 8-item health-related quality of life instrument (HINT-8) subfactors according to the multiple logistic regression analysis. Adjusted for age, sex, household income, education level, marital status, employment status, current smoking status, and current drinking status.

Subfactors of HINT-8 and latent class^a^	Class 1 (relatively healthy), OR^b^ (95% CI)	Class 2 (cardiometabolic conditions), OR (95% CI)	Class 3 (arthritis, allergy, or asthma), OR (95% CI)
Difficulty in climbing stairs	1 (reference)	1.49 (1.08-2.06)	1.92 (1.35-2.71)
Pain	1 (reference)	1.58 (1.13-2.23)	1.88 (1.32-2.67)
Lack of vitality	1 (reference)	1.07 (0.83-1.37)	1.35 (1.02-1.79)
Difficulty in working	1 (reference)	1.22 (0.87-1.72)	1.28 (0.56-2.91)
Depression	1 (reference)	1.34 (0.57-3.94)	2.51 (1.09-4.98)
Difficulty in memory	1 (reference)	0.98 (0.46-1.92)	1.02 (0.32-1.99)
Sleep problems	1 (reference)	1.07 (0.54-1-31)	1.46 (1.01-2.10)
Unhappiness	1 (reference)	1.28 (1.03-1.69)	1.54 (1.13-2.26)

^a^Multiple logistic regression analysis was performed by classifying the dependent variable as binary.

^b^OR: odds ratio.

## Discussion

### Principal Findings and Comparison With Previous Work

This study investigated the prevalence of multimorbidity, chronic disease patterns, and the association between latent classes and quality of life among Korean older adults using LCA. Of the sample of 1806 Korean older adults, 54.8% had multimorbidities, with a mean number of chronic conditions of 1.82 (SD 1.35). This prevalence of multimorbidity was lower than that found by previous studies in other countries. At least two-thirds of those older than 65 years have multimorbidities in the United Kingdom and the United States [22,23], while approximately 80% of Australians aged 65 years and older have 3 or more chronic conditions [8]. The standardized national prevalences of multimorbidity in Japan are approximately 50% for ages between 60 and 69 years and 60% for ages between 70 and 74 years [24].

Using LCA, this study identified 3 chronic disease groups: a relatively healthy group without multimorbidities, a cardiometabolic condition group, and an arthritis, allergy, or asthma group. The cardiometabolic condition group had a high probability of having hypertension, dyslipidemia, diabetes mellitus, myocardial infarction, and stroke, while the other group had a high probability of experiencing arthritis, osteoarthritis, allergic rhinitis, or asthma. Similar disease patterns to those found in this study have been noted previously [3,25-28]. Cardiometabolic diseases are one of the most reproducible multimorbidity patterns [29]. The association between dietary patterns and multimorbidity within the cardiometabolic domain suggests the importance of dietary interventions in preventing and managing multimorbidity [30].

Our findings were also similar to the results of another study among Koreans aged 50 years and older that classified hypertension, hyperlipidemia, diabetes, and stroke as a single latent class of multimorbidity using data from the KNHANES conducted in 2013 and 2014 [3]. Another study using a network analysis approach indicated that hypertension had stronger connections with hyperlipidemia and diabetes than other pairs of morbidities in older adults, using data from the KNHANES conducted between 2010 and 2018. The substantial overlap between hypertension, diabetes, and hyperlipidemia in etiology and disease mechanisms might have resulted in this phenomenon [2]. The further replicability of results between studies that use different methodologies is an important step toward moving from exploratory to confirmatory approaches.

This study indicated that the association between chronic disease patterns and HRQoL differed by multimorbidity group. The cardiometabolic condition and arthritis, allergy, or asthma groups had lower HRQoL than the relatively healthy group [31-34]. Moreover, the total scores of the HINT-8 were higher in the arthritis, allergy, or asthma group than in the cardiometabolic condition group, indicating a lower quality of life [35].

Compared with older adults with a single chronic disease, those with multiple chronic conditions face mental health-related social needs and social isolation [36,37]. Their low quality of life and combination of chronic diseases may diminish their overall health [28]. Thus, these chronic disease patterns can be used to support better screening, targeted management, and integrated health services for older adults with chronic diseases [32,38,39]. Further research is required to investigate whether multimorbidity associations are similar to biological pathways or the result of interactions.

Current health care models are disease-oriented, meaning that the management of chronic conditions applies to a single condition and may not be relevant to those with multimorbidities [40]. Individuals with multimorbidities typically receive services from multiple health care specialists who only focus on a single health condition and do not view them in their entirety. A key challenge faced by individuals with multimorbidities, and health care professionals is thus fragmented clinical care, which creates miscommunication; this may result in inconsistent health care messages and could lead to an increase in polypharmacy [28]. Consequently, further research is needed to develop guidelines and approaches for the management of multimorbidity and identify chronic disease groups.

### Limitations

This study had some limitations. First, it focused on a limited range of chronic diseases, specifically those with a prevalence of at least 3% as reported by the KNHANES; hence, the list of chronic conditions may not have been exhaustive. If more diseases are considered, a higher prevalence could be estimated [41]. However, while we performed an additional LCA including all the chronic diseases registered in the KNHANES data set, the samples for certain combinations of multimorbidities were insufficient to provide reliable results despite our relatively large sample overall [9].

Second, the findings could differ if a broader scope of chronic conditions is considered along with the different aspects of the conditions. A long illness negatively impacts the quality of life of chronic patients [42]. Moreover, information on disease severity may have helped distinguish between patterns; unfortunately, these data were unavailable.

Third, the participants self-reported their chronic diseases, increasing the risk of reporting bias. However, self-reported data are the most appropriate alternative in the absence of objective diagnoses and have been shown to provide solid estimates [9,43].

Finally, the selected class name in LCA may not always accurately represent class membership [20]. However, any issues arising from this so-called “naming fallacy” may be mediated by presenting the distribution of the selected indicators for each class (Table 1) to allow an individual interpretation. Despite these limitations, the chronic disease patterns among older adults were examined using nationally representative data.

### Conclusions

This study identified 3 distinct chronic disease groups among Korean older adults: a relatively healthy group without multimorbidities, a cardiometabolic condition group, and an arthritis, allergy, or asthma group. Those in the latter group had a lower quality of life than those in the cardiometabolic condition group. Understanding these combinations of long-term conditions may help target disease prevention and improve care integration efforts. The further replicability of results between studies that use different methodologies is an important step toward moving from exploratory to confirmatory approaches. Future research on the effectiveness of interventions aimed at preventing and improving the management of multimorbidities is needed.

## Appendix

Multimedia Appendix 1 Latent class model identification and model fit statistics and elbow plot of the latent class model fit.

## Abbreviations

HINT-8: 8-item health-related quality of life scale
HRQoL: health-related quality of life
IRB: institutional review board
KNHANES: Korean National Health and Nutritional Examination Survey
LCA: latent class analysis
OR: odds ratio

## Data Availability

The data sets generated during and/or analyzed during this study are available in the Korean National Health and Nutritional Examination Survey website repository.
