# Supplementary material for: Chronic Disease Patterns and Their Relationship With Health-Related Quality of Life in South Korean Older Adults With the 2021 Korean National Health and Nutrition Examination Survey: Latent Class Analysis
Source: JMIR Public Health Surveill. 2024 Apr 10;10:e49433. doi: 10.2196/49433 (PMC11043926; doi:10.2196/49433)
Supplement: Multimedia Appendix 1 [file publichealth_v10i1e49433_app1.docx]

**Table S1**. Latent class model identification and model fit statistics.

| **Class** | **LL** | **AIC** | **CAIC** | **BIC** | **Entropy** | **df** | **G²** | **p** |
| --- | --- | --- | --- | --- | --- | --- | --- | --- |
| 2 | –7194 | 14433 | 14782 | 14759 | 0.810 | 1875 | 617 | 0.040 |
| **3** | –**7146** | **14362** | **14589** | **14554** | **0.876** | **1763** | **522** | **0.012** |
| 4 | –7126 | 14345 | 14651 | 14604 | 0.849 | 1751 | 481 | 0.058 |
| 5 | –7116 | 14350 | 14733 | 14674 | 0.826 | 1739 | 462 | 0.360 |

Note. Bold rows represent the identified model. AIC: Akaike information criterion, BIC: Bayesian information criterion; CAIC: Consistent Akaike information criterion, LL: Log-likelihood, p: bootstrap p-value.

**Figure S1**. Elbow plot of the latent class model fit.

Note. AIC: Akaike information criterion, BIC: Bayesian information criterion, CAIC: Consistent Akaike information criterion
